# Differential response of the thyroid axis to high-fat diet-induced inflammation and energy balance deregulation in juvenile and adult rats

**DOI:** 10.3389/fendo.2025.1676754

**Published:** 2025-11-21

**Authors:** Elena Alvarez-Salas, Paulina Soberanes-Chávez, Alicia Andrade, Juan Luis Dolores-SanJuan, Cinthia García-Luna, Gilberto Matamoros-Trejo, Patricia de Gortari

**Affiliations:** 1 Laboratorio de Neurofisiología Molecular, Instituto Nacional de Psiquiatría Ramón de la Fuente Muñiz, Mexico City, Mexico; 2 Escuela de Dietética y Nutrición del ISSSTE, Mexico City, Mexico

**Keywords:** high-fat diet, thyroid axis, interleukin-1β, neuroinflammation, hypothalamus

## Abstract

**Introduction:**

The hypothalamic–pituitary–thyroid (HPT) axis regulates metabolic rate and adapts to fluctuations in energy demand, through changing the expression of thyrotropin releasing hormone (TRH) in the hypothalamic paraventricular nucleus (PVN), which drives those neuroendocrine responses. TRHergic neurons integrate multiple signals, including those arising from infections and dietary energy challenges. High-fat diet (HFD) intake, for example, activates PVN TRH mRNA and the HPT axis to manage energy excess; also, it promotes interleukin-1β (IL-1β)-mediated neuroinflammation disrupting axis function. How HPT axis adapts to simultaneous metabolic and inflammatory stimuli across development and under different durations of HFD exposure remains unelucidated.

**Methods:**

We investigated these interactions in juvenile (21 days old) and adult (2.5 months old) Wistar rats exposed to HFD for 4 or 12 weeks.

**Results:**

Our findings showed that the longer the HFD exposure and the younger the animals, the greater the impairment of HPT axis function and body weight (b.w.) regulation. Short-term HFD feeding revealed age-dependent effects on b.w. since only juveniles were heavier than their controls, while the HPT axis was active on both. Neuroinflammatory responses also differed by developmental stage and duration. After 4 weeks of HFD, only juveniles displayed elevated IL-1β protein expression, whereas after 12 weeks, both age groups exhibited increased IL-1β; however, adults remained more resilient to cytokine overexpression than juveniles, regarding HPT axis function.

**Discussion:**

Prolonged exposure promoted IL-1β–associated neuroinflammation, altered HPT axis regulation and favored leptin resistance, particularly in juveniles. These results highlighted the critical role of developmental stage and diet duration in HFD intake neuroendocrine effects.

## Introduction

The hypothalamic-pituitary-thyroid (HPT) axis is a neuroendocrine system that regulates energy metabolism in humans and animals. By modulating lipid reserve mobilization, it integrates signals and adapts to changing energy demands posed by environmental stressors, nutritional status, and illness, helping individuals to survive under challenging conditions (1, 2).

Thyrotropin-releasing hormone (TRH), synthesized in the medial parvocellular region of the hypothalamic paraventricular nucleus (PVN), is the neuropeptide controlling HPT axis activity. Upon its release into the median eminence, TRH binds to TRH-R1 receptors expressed on pituitary thyrotropes and stimulates the release of thyroid-stimulating hormone (TSH) to circulation. TSH then acts on the thyroid gland increasing the synthesis and release of the thyroid hormones (TH) thyroxine (T_4_) and triiodothyronine (T_3_) (3). T_3_, the biologically active form, enhances energy availability by stimulating lipolysis in the adipocytes and thermogenesis. Through these mechanisms, the HPT axis enables animals to respond effectively to metabolic and environmental challenges by increasing energy expenditure when highly available, which avoids lipid accumulation (4).

Metabolic conditions such as overweight and obesity or pro-inflammatory responses to infection, modulate HPT axis activity by increasing or decreasing hypothalamic TRH expression and release, respectively (1, 5). When positive energy balance becomes chronic (e.g., over 6–10 weeks), lipid mobilization increases as a compensatory mechanism preventing excessive weight gain and obesity (6). This adaptation involves an alteration in the HPT axis regulation, since the negative feedback mechanism is blunted during HFD exposure: despite elevated T_3_ and TSH serum concentrations, TRH mRNA expression in the PVN does not decrease but instead increases (7, 8). Such upregulation enhances energy utilization and contributes to maintaining energy homeostasis (9).

In contrast, inflammatory processes typically decelerate the HPT axis activity. Proinflammatory cytokines such as interleukin (IL)-6 and IL-1β produced by activated hypothalamic microglia, downregulates TRH expression in the PVN or adenohypophyseal TSH release, depending on the etiology of the inflammatory stimulus (10–14).

TRH synthesis may be modulated directly or indirectly through various cytokines-activated intracellular pathways. For example, IL**-**1β can activate NF-kB signaling in the third ventricle lining tanycytes, which increases type 2 deiodinase activity and consequently the local T_3_ content (9, 15). This T_3_ rise acts on TRHergic neurons, reduces TRH transcription (16) and suppresses HPT axis activity, an effect that persists as long as the inflammatory process remains active (12, 17). These observations indicate that multiple components of HPT axis regulation can be targeted for effector molecules of physiological or pathological conditions that impose diverse energy demands.

High-fat diet (HFD) intake is of particular interest because it induces both: neuroinflammation (which tends to suppress TRH expression) and increased lipid accumulation (which typically avoids a PVN TRH downregulation), thus the resulting HPT axis modulation is intriguing and unpredictable.

Animal responses to HFD-induced peripheral and central alterations vary with age: i.e. access to HFD during the juvenile period, but not in adulthood, may disrupt glucose and lipid metabolism by the neuroinflammation-induced impairments in HPT axis functioning. It could also be associated to the negative and risky feeding behavioral patterns that adolescents are prone to develop by increased cytokine levels or other inflammatory factors in brain (18–20).

Thus, the adaptation of the HPT axis and its consequences in development when various effectors impinge on TRHergic neurons, depends on the brain maturation and HPT axis ability to integrate and prioritize competing intracellular signals to maintain energy homeostasis.

In this study, we examined changes in TRH gene and protein expression in the PVN and medialbasal hypothalamus (MBH), respectively; also, TSH and TH blood concentrations in juvenile (21-day-old) and adult (2.5-month-old) rats fed either with a standard diet or a HFD for 4 or 12 weeks. We also monitored weekly body weight (b.w.) and daily food intake, evaluated PVN IL-1β gene expression, as well as its content in the MBH (that includes arcuate nucleus), and serum leptin and corticosterone levels as potential modulators of HPT axis activity.

Our rationale is supported by evidence showing that both, duration of HFD intake and age of animals, are critical factors for shaping HPT axis adaptive response to changes in energy demand (21), and to neuroinflammation-induced alterations. We focused on IL-1β as a potential key effector of neuroinflammation in the hypothalamus, given its role in stimulating appetite-regulating peptides such as, the α-melanocyte-stimulating hormone (α-MSH) in the arcuate nucleus, which in turn can modulate TRH expression in the PVN (22).

Overall, our work aims to clarify how TRH integrates signals from positive energy balance and HFD-induced neuroinflammation, advancing in the understanding of neuroendocrine adaptive mechanisms that ultimately may impact on the lipid accumulation extent of animals and in their susceptibility to obesity development and other associated metabolic alterations.

## Materials and methods

### Animals

All procedures were conducted with the approval of the local Ethics Committee on Animal Experimentation of the National Institute of Psychiatry (INPRFM) and in compliance with the Mexican Official Norm NOM-062-ZOO-1999.

We used 18 juvenile male Wistar rats (postnatal days 21-23) and 22 adults (2.5 months of old) from the *vivarium* of the INPRFM. The animals were pair-housed and maintained under controlled conditions on a 12/12-hour light/dark cycle (lights on at 7:00) and acclimatized for three days before the commencement of the experiment.

**Figure 1:**
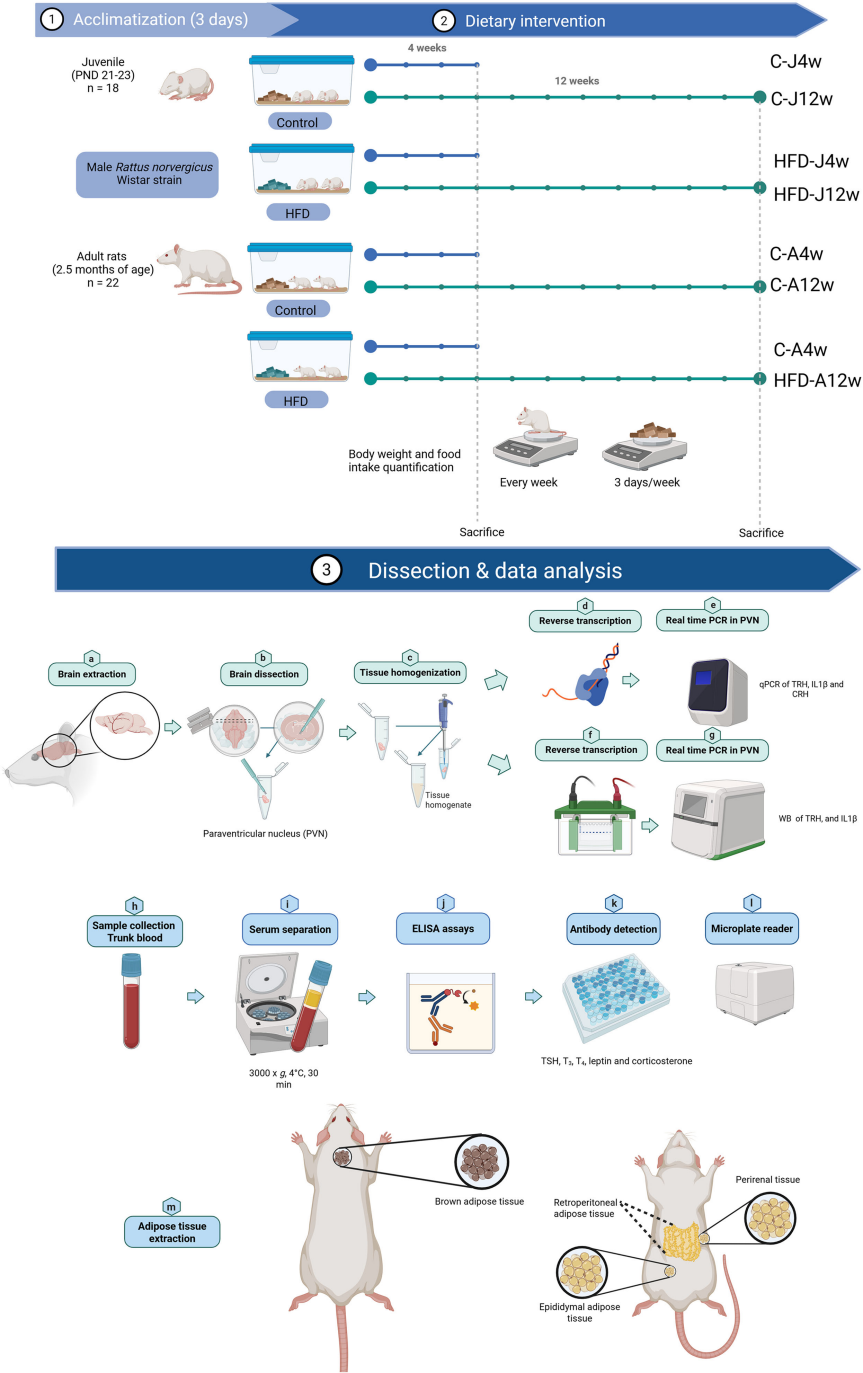
Experimental design for both juvenile and adult rats involved exposure to a HFD for either 4 or 12 weeks. 1. Male rats were housed in pairs during the acclimation period (3 days). 2. They were divided into eight experimental groups: juvenile and adult rats with either a control or HFD for 4 or 12 weeks (C-J4w, HFD-J4w, C-J12w, HFD-J12w, C-A4w, HFD-A4w, C-A12w and HFD-A12w, n=4-6/group). Throughout the experiment, body weight was measured weekly, and food intake recorded three days per week. 3. After 4- or 12-weeks animals were euthanized by decapitation, and their brains were extracted and cryopreserved for subsequent quantification of genes of interest by RT-PCR in the PVN (a-g). Trunk blood was collected, and serum was obtained for hormone determinations: TSH, T_3_, T_4_, leptin, and CORT, using ELISA assays (h-l). Different adipose tissue depots were excised: representation of adipose tissue sectioning, encompassing brown, perirenal, epididymal, and retroperitoneal adipose tissues (m). Postnatal day (PND), high-fat diet (HFD), hypothalamic paraventricular nucleus (PVN), thyroid stimulating hormone (TSH), triiodothyronine (T_3_), thyroxine (T_4_), corticosterone (CORT). Created with BioRender.

Animals were randomly assigned to control (C) or HFD groups. HFD groups were fed with a commercial diet containing 60% Kcal from fat, 20% Kcal from protein and 20% of Kcal from carbohydrate (D12492; energy density of 5.2 Kcal/g), whereas control animals were offered a diet containing 10% Kcal from fat, 20% Kcal from protein and 70% Kcal of carbohydrate, matched in sucrose content (D12450J; energy density of 3.8 Kcal/g) (Research Diets, New Brunswick, NJ). Diets were administered for 4 or 12 weeks, forming eight groups defined by age, diet type, and diet-exposure duration (n=4-6/group) (**Figure 1**). Water and chow were *ad libitum* supplied. B.w. was registered weekly and food intake was quantified 3 days/week by subtracting the weight of uneaten food from the amount offered on the previous day, then dividing the amount of food by the number of animals per cage.

### Adipose tissue collection

Rats were euthanized by decapitation performed by a highly trained technician to minimize animal distress, following the American Veterinary Medical Association (AVMA) Guidelines for the Euthanasia of Animals (2020). Adipose tissue was dissected and weighed. Fat depots were carefully collected from perirenal (around the kidneys and adrenal glands in the retroperitoneal space), retroperitoneal (behind the abdominal or peritoneal cavity), epididymal (a narrow, tightly coiled tube attached to the testes) and brown adipose tissue (BAT) located behind the neck. The adiposity index was calculated as previously reported (23), by adding the weight of all white fat depots (excluding BAT), dividing by final b.w., and multiplying by 100 to yield a percentage reflecting overall adiposity. This index serves as an indicator of obesity in animals.

### Blood collection and ELISA assays

Following decapitation (between 9:00-11:00h), trunk blood was collected and centrifuged at 3,000 x *g* for 30 minutes at 4°C to extract serum. Serum samples were aliquoted and stored at -70°C until further analysis. Serum samples were processed in duplicate using commercially available enzyme-linked immunosorbent assay (ELISA) kits, following manufacturer instructions.

We used 25 μL of serum for TSH measurement (ALPCO, Salem, NH); sensitivity: 0.081 ng/mL; inter and intra-assay variation: 3.4% and 6. 3%, respectively; 70 μL (1:2 dilution) and 25 μL of serum for total triiodothyronine (T_3_) and total thyroxine (T_4_) levels respectively (ALPCO); sensitivity: 0.1 ng/mL (T_3_) and 0.5 μg/dL (T_4_); inter-assay variability: 10.3% (T_3_) and 4.5% (T_4_); intra-assay variability: 9.6% (T_3_) and 4.3% (T_4_). Leptin and corticosterone (CORT) were analyzed using 60 μL (1:4 dilution) and 50 μL (1:50 dilution), respectively (Enzo Life Sciences Farmingdale, NY); sensitivity: 67.2 pg/mL (leptin) and 27 pg/mL (CORT); inter-assay variability: 6.5% (leptin) and 8.2% (CORT); intra-assay variability: 7.1% (leptin) and 8.4% (CORT). A μQuant microplate reader (BioTek Instrument Inc, Winooski, VT) and BioTek’s Gen5 Data Analysis Software (BioTek ELx800, Fisher HealthCare) were used to determine hormone levels.

### qRT-PCR

The frozen PVN was extracted from coronal brain slices located between 0.84 mm and 2.04 mm from bregma (24), using a 1 mm diameter sample corer. Samples were homogenized in 4 M guanidine thiocyanate. Total RNA was extracted according to standard procedures (25); the assessment of RNA sample quality entailed the measurement of O.D. absorbances at 260/280 and 260/230 nm. Samples with a ratio below 1.5 were excluded from further analysis. For cDNA synthesis, 1.5 μg of RNA and oligo dT (100 pmol/ml; Biotecnologías Universitarias, Universidad Nacional Autónoma de México, México) were used, along with M-MLV reverse transcriptase (Invitrogen Waltham, MA). We conducted a reverse transcription polymerase chain reaction (RT-PCR) to quantify TRH and IL-1β mRNA levels in the PVN. RT-PCR was conducted using TaqMan Universal PCR Master Mix (ThermoFisher Scientific, Waltham, MA) on a QuantStudio 3 system (ThermoFisher) with the following probes: *Trh* (Rn00564880_m1), *IL1B* (Rn00580432_m1) (ThermoFisher). Each PCR reaction was performed in duplicate. mRNA expression levels were normalized to those of the housekeeping gene *Actb* (Rn00667869_m1). The assessment of mRNA content changes in the PVN tissue was conducted using the ΔCT method. The ΔCT value was calculated as the difference between the cycle threshold (Ct) values for each gene of interest and *Actb* (ΔCt = Ct of target gene-Ct *Actb*). The groups used as references were the control groups at 4 and 12 weeks. The fluorescence value for the control group, expressed in arbitrary units, was set to 1. The percentage of change for each examined gene was calculated using the following equation: _ΔΔCt = 2_-(ΔCt=Ct Control-Ct experimental group).

### Immunoblotting

The MBH was hand-dissected from a thick coronal brain slice (approximately −0.6 to −3.6 mm from bregma; H 8.2 to 9.8 mm; L −1.0 to 1.0 mm) containing the median eminence and the arcuate nucleus and was used to measure TRH and IL-1β content. A total of 80 µL of RIPA lysis buffer per tissue sample (Abcam, Cambridge, UK) with protease inhibitor (1:100, Thermo Fisher Scientific, Waltham, MA, USA) was used to extract proteins using a sonicator. Tissue lysates were incubated for 20 min on ice and then centrifuged at 13,800 rpm at 4 °C for 10 min. Supernatants were collected, the same volume of 2X Laemmli buffer (Merck, Darmstadt, Germany) was added, and the samples were denatured at 95°C for 5 min.

Protein determination was performed by the micro-Lowry method. Protein samples (10 µg) were loaded in 14% SDS-PAGE gels for electrophoresis and, after 1.5 h, were transferred to Hybond-C extra nitrocellulose membranes (Amersham, Life Science, Buckinghamshire, UK). The membranes were then incubated in a blocking solution of 5% BSA in 1X PBS/Tween 0.1% for 24 h and for 48 h with the primary antibodies for TRH (1:1K, rabbit monoclonal anti-TRH, cat. ab171958, Abcam, Waltham, MA, USA) and IL1β (1:2K rabbit polyclonal anti-IL1-β, cat. ab205924 Abcam) diluted in the blocking solution. After washing with PBS/0.1% Tween, the membranes were incubated for 1 h with the secondary antibody (1:10K goat anti-rabbit HRP conjugate, cat. ab6721 Abcam) in blocking solution. The membranes were then incubated with the polyclonal mouse anti-β actin antibody (1:1K, cat. sc-47778, Santa Cruz Biotechnology, Inc., Dallas, TX) as a loading control using donkey anti-mouse HRP (1:10K cat. 715-035–150 Jackson Immunoresearch, West Grove, PA, USA) as the secondary antibody. Protein bands were revealed using luminol and visualized with a densitometer iBright CL1000 imaging system (Invitrogen). ImageJ software was used for the analysis and quantification of luminescent signals, and the values are the intensity of the TRH/actin or IL-1β/actin signals in arbitrary units.

### Statistics

Data are expressed as the mean ± standard error of the mean (SEM). Normality was assessed using the Shapiro–Wilk test. B.w. gain, adipose tissue weight and food intake (normalized by b.w. and expressed as Kcal/Kg b.w) were analyzed using two-way repeated measures ANOVA. Hormone levels and mRNA expression were analyzed using two-way ANOVA, with diet type (control or HFD) and duration of exposure (4 or 12 weeks) as factors. Tukey’s *post hoc* test was conducted when p<0.05. Protein content was analyzed using Student’s *T*-test between C and HFD groups. If the normality test failed a non-parametric test was used. All statistical analyses were performed using Prism version 8.0.2 (GraphPad Software, San Diego, CA, USA).

## Results

### Body weight gain

B.w. gain was significantly higher in both juvenile groups exposed to the HFD compared to their respective controls. HFD-J4w rats showed a 19% increase in b.w. gain. A two-way repeated measures ANOVA showed an effect of time (F_(3,30)_ = 810.2, p<0.001), diet (F_(1,10)_ = 10.4, p<0.05) and an interaction between diet and time (F_(3,30)_ = 4.6, p<0.05). In the HFD-J12w group, b.w. gain increased by 20% vs. control animals, with significant effects of time (F_(11,77)_ = 447.2, p<0.001) and an interaction between diet and time (F_(11,77)_ = 4.4, p<0.001).

**Figure 2:**
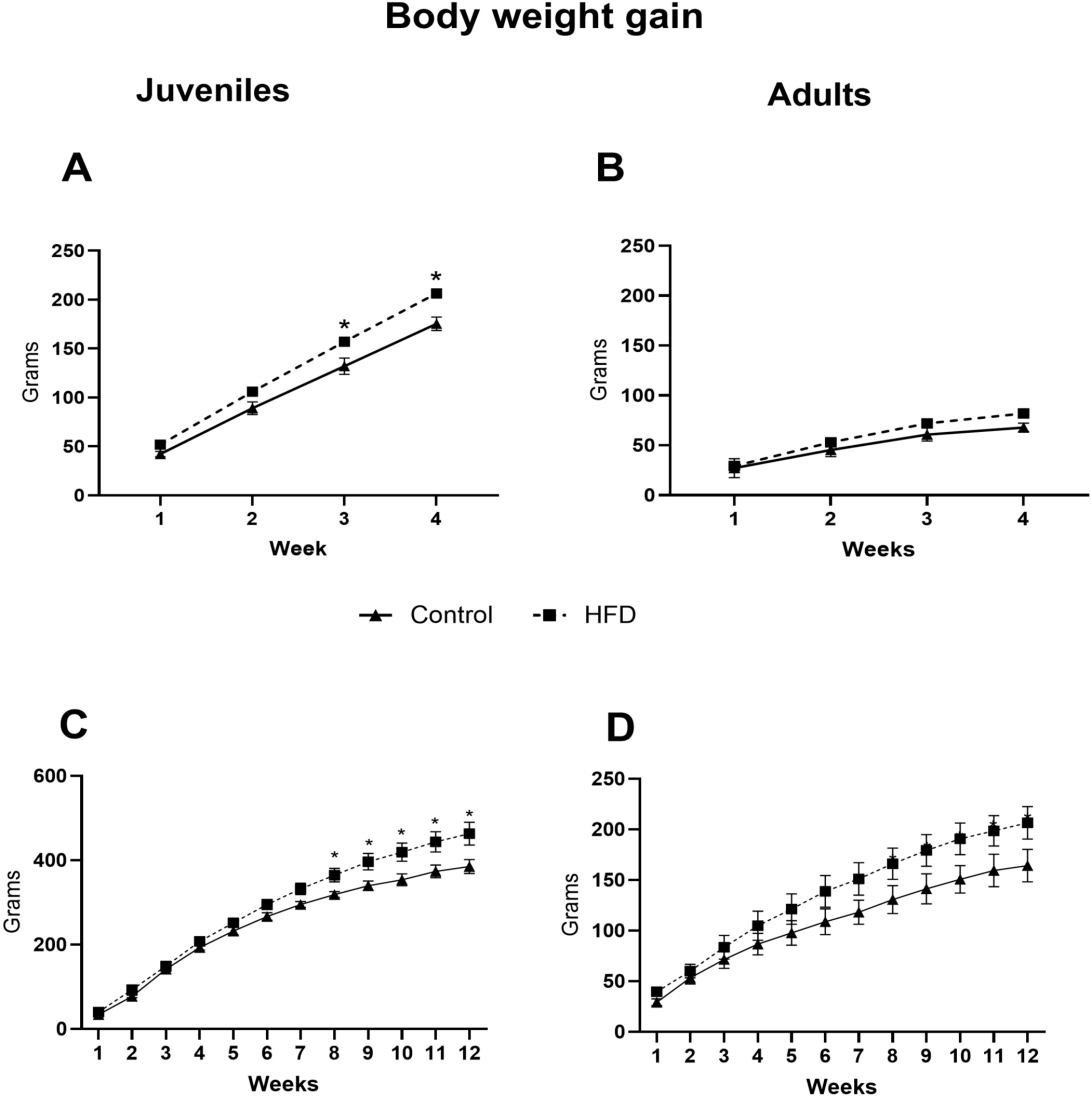
Body weight gain over time in juvenile and adult rats. The data reflect the body weight variations in male juvenile **(A, C)** and adult **(B, D)** rats fed either a standard control diet or a high-fat diet (HFD) during 4 or 12 weeks. n=4-6rats/group. *p<0.05 vs. the control group.

In adults, b.w. gain was not affected by 4 weeks of a HFD (HFD-A4w), although a significant effect of time was observed (F_(3,18)_ = 107.5, p<0.001). However, after 12 weeks of HFD exposure, adult rats exhibited a 26% increase in b.w. gain compared to controls. The two-way repeated measures ANOVA showed a significant effect of time (F_(11,77)_ = 123, p<0.001) and an interaction between diet and time (F_(11,77)_ = 2.05, p<0.05) (**Figures 2A–D**).

### Adiposity levels

**Figure 3:**
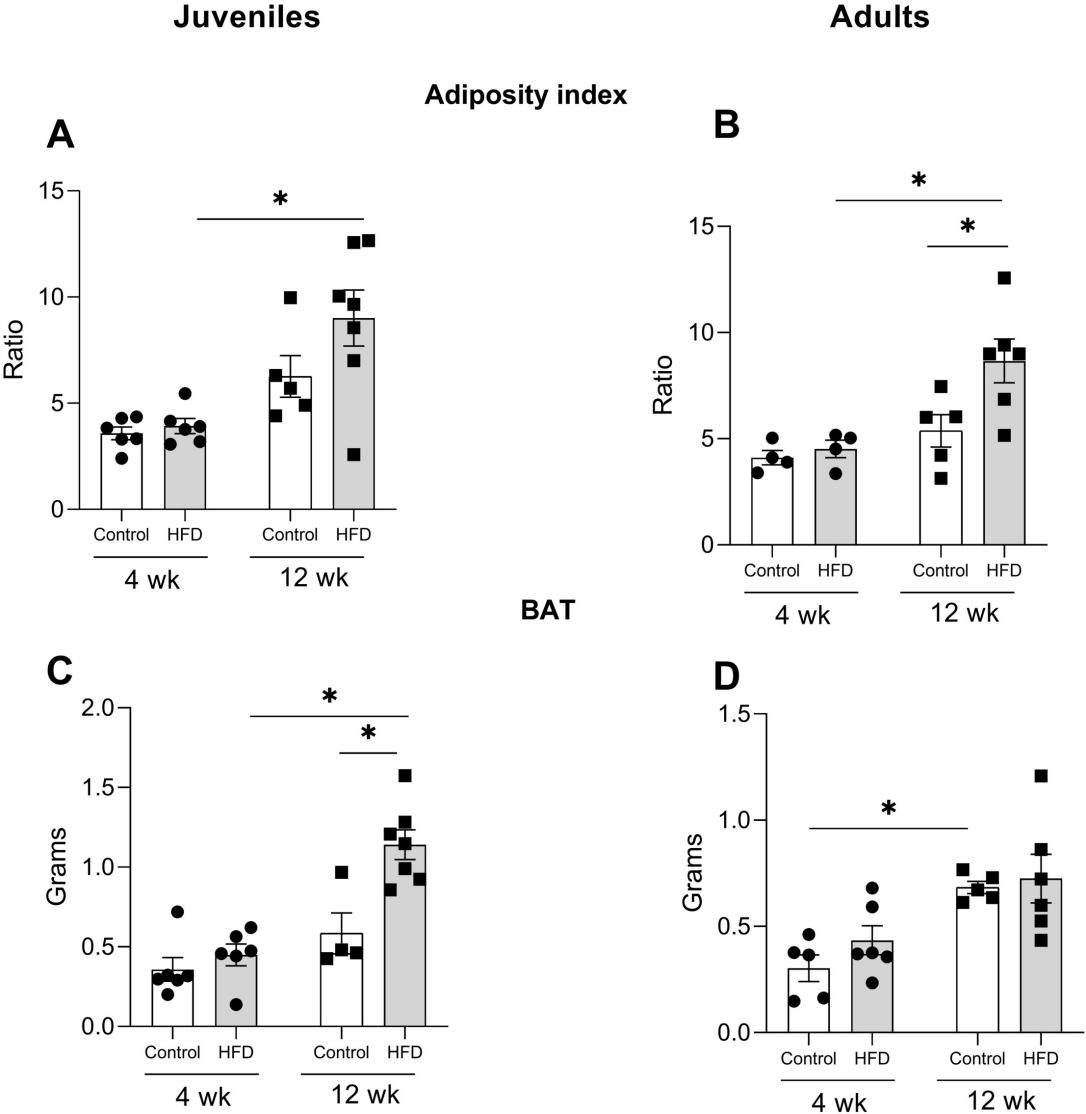
Quantification of adiposity index in juveniles **(A)** and adults **(B)**. BAT is depicted in panels **(C)** (juvenile rats) and **(D)** (adult rats). Data presented as the mean ± S.E.M and is expressed in grams. n=4–6 rats/group. *p<0.05 between depicted groups. The adiposity index was calculated by adding the weight of all fat depots, excluding brown adipose tissue (BAT), dividing this total by the final body weight, and multiplying by 100.

An analysis of the adiposity index in the visceral compartments and the weight of BAT was conducted to better understand the specific impact of the HFD on fat mass (**Figure 3**). The data revealed that HFD-J12w exhibited a statistically significant increase of 130% in adiposity, when compared to those exposed to the same diet for 4 weeks (HFD-J4w) (**Figure 3A**). In adult rats, the adiposity index was 61% higher in HFD-A12w compared to their controls and 92% higher than that of the HFD-A4w group (**Figure 3B**). The two-way ANOVA showed significant differences in juvenile rats based on the factor time (F_(1,20)_ = 18.05, p<0.001). In adults, significant effects were observed for both diet (F_(1,15)_ = 5.096, p<0.05) and time (F_(1,15)_ = 10.86, p<0.001).

BAT weight changed differently: juvenile rats exposed 4 w to a HFD had a 154% increase in BAT weight, while after 12 weeks (HFD-J12w) the increase was 95% when compared to animals on a control diet. (**Figure 3C**). Two-way ANOVA showed significant effects of diet (F_(1,19)_ = 12.68, p<0.05), time (F_(1,19)_ = 25.37, p<0.001), and the interaction between these factors (F_(1,19)_ = 6.532, p<0.05). Adult rats showed no significant changes in BAT weight (**Figure 3D**).

### Food intake

No significant differences in caloric intake (Kcal/Kg) were observed across age or durations of the experiment (**Tables 1**, **2**).

**Table 1:** Energy intake of juvenile and adult rats fed a standard or high fat diet over 4 weeks.

Kcal/Kg					
Juveniles	Group	1w	2w	3w	4w
	Control	423.8 ± 13.4	385.6 ± 14.4	335.9 ± 7.7	291.6 ± 7.7
	HFD	403.8 ± 41.1	378.9 ± 20.6	346.4 ± 11.3	317.3 ± 14.9
Adults		1w	2w	3w	4w
	Control	246.4 ± 12.6	225.2 ± 16.6	209.4 ± 12.8	184.6 ± 5.7
	HFD	242.8 ± 13.3	224.2 ± 12.9	220.6 ± 15.1	215.5 ± 15.4

Energy intake of juvenile and adult rats exposed to a standard (control) or to a high-fat diet (HFD) over 4 weeks. Data are presented as the mean ± S.E.M. and expressed in Kcal/ Kg of body weight, n=4–6 rats/group.

**Table 2:** Energy intake of juvenile and adult rats fed a standard or high fat diet over 12 weeks.

Kcal/Kg					
Juveniles	Group	1w	4w	8w	12w
	Control	375.7 ± 42.0	329.7 ± 8.5	229.6 ± 8.5	189.2 ± 10.7
	HFD	450.3 ± 23.4	366.1 ± 23.4	255.9 ± 21.4	218.6 ± 20.0
Adults	Group	1w	2w	3w	4w
	Control	272 ± 13.4	213.8 ± 10.6	189.0 ± 8.2	186.9 ± 13.1
	HFD	278.9 ± 12.5	224.3 ± 13.5	214.7 ±13.4	180.8 ± 11.5

Energy intake of juvenile and adult rats exposed to a standard (control) or to a high-fat diet (HFD) over 12 weeks. Data are presented as the mean ± S.E.M. and expressed in Kcal/Kg of body weight, n=4–6 rats/group.

### HPT axis function

**Figure 4:**
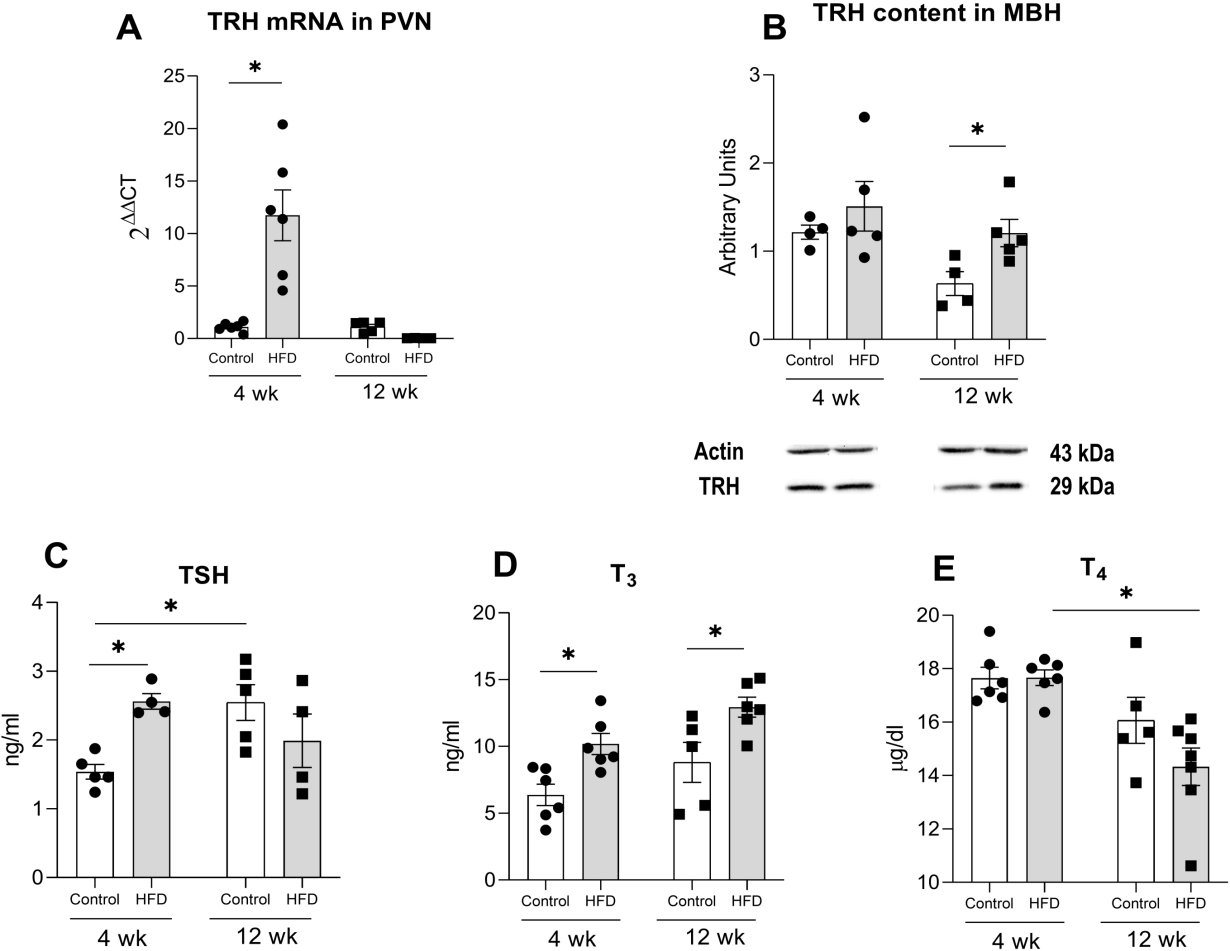
Effects of high fat diet (HFD) consumption on components of the thyroid axis in juvenile rats. **(A)** TRH mRNA expression in the PVN. **(B)** TRH protein content in the medial basal hypothalamus (MBH). **(C)** Serum levels of thyroid-stimulating hormone (TSH) **(D)** triiodothyronine (T_3_) and **(E)** thyroxine (T_4_). Data presented as the mean ± S.E.M. (n=4-6/group). *p<0.05 between depicted groups.

**Figure 5:**
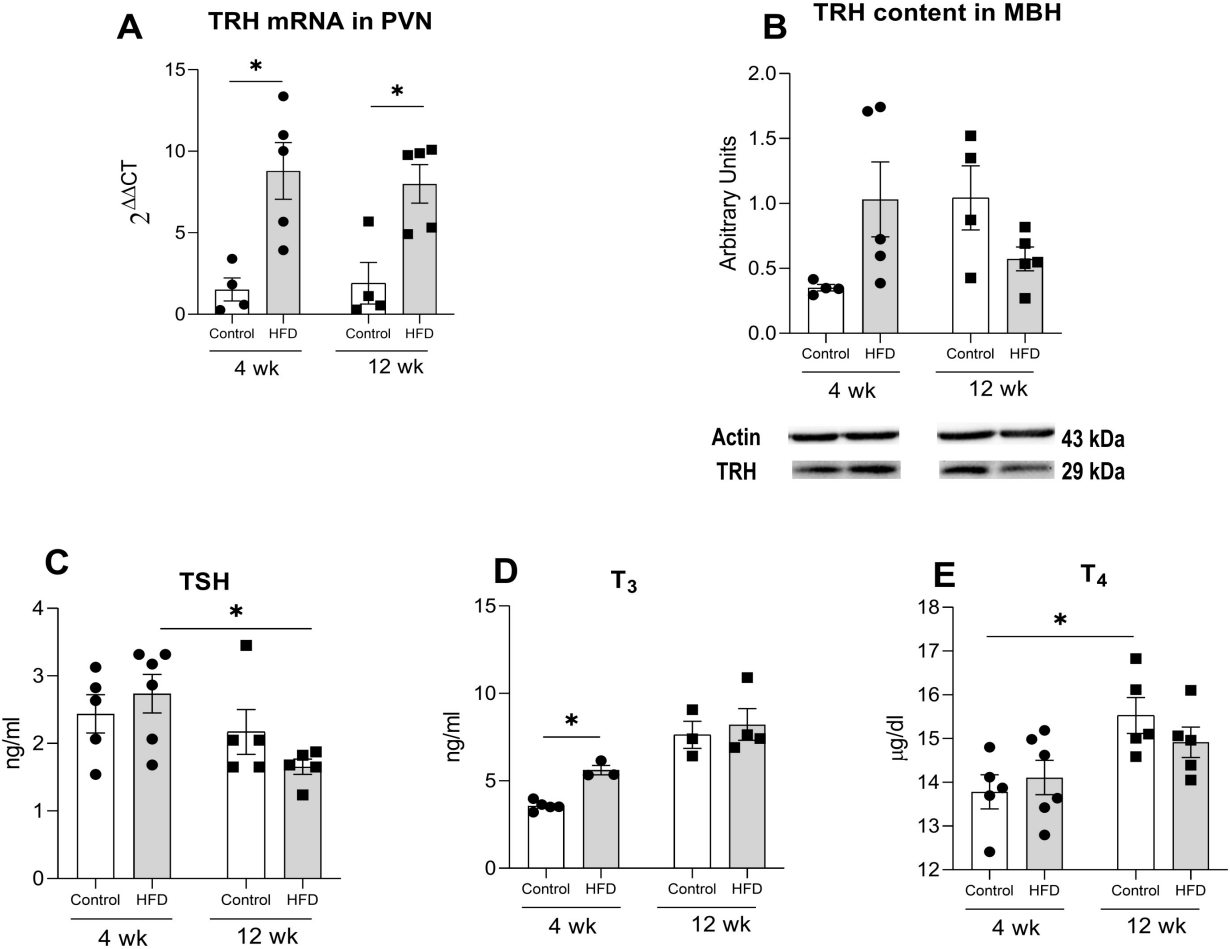
Effects of high fat diet (HFD) consumption on components of the thyroid axis in adult rats. **(A)** TRH mRNA expression in the PVN. **(B)** TRH protein content in the medial basal hypothalamus (MBH). **(C)** Serum levels of thyroid-stimulating hormone (TSH) **(D)** triiodothyronine (T_3_) and **(E)** thyroxine (T_4_). Data presented as the mean ± S.E.M. (n=4-6/group). *p<0.05 between depicted groups.

Juvenile rats showed distinct patterns of TRH mRNA expression in the PVN: levels increased 10-fold in the HFD-J4w group but decreased by 70% in HFD-J12w compared to their respective control groups (**Figure 4A**). Two-way ANOVA revealed significant main effects of diet (F_(1,17)_ = 11.13, p<0.01) and intake duration (F_(1,17)_ = 16.65, p<0.001), as well as a significant interaction between these factors (F_(1,17)_ = 16.80, p<0.001). In adult rats, HFD feeding led to increased PVN TRH mRNA expression at both 4 and 12 weeks (**Figure 5A**), with two-way ANOVA showing a significant main effect of diet (F_(1,14)_ = 24.41, p<0.001).

After 4 weeks of HFD exposure, TRH protein levels in the MBH remained unchanged in both juvenile and adult groups (**Figures 4B**, **5B**). In contrast, after 12 weeks, HFD significantly increased TRH content by nearly two-fold in juveniles (t(7) = 2.07 p< 0.05), whereas adults showed a non-significant trend toward reduced TRH levels compared to controls (t(7)= 1.5 p = 0.07) (**Figures 4B**, **5B**).

The HFD-J4w group showed higher TSH levels than C-J4w (**Figure 4C**); two-way ANOVA showed a significant interaction between diet and time (F_(1,14)_ = 10.9, p<0.01). HFD-fed juvenile animals exhibited a similar increase in T_3_ levels compared to controls, regardless of the duration of diet exposure (**Figure 4D**). A two-way ANOVA showed a significant effect of diet (F_(1,19)_ = 17.1, p<0.001) and time (F_(1,19)_ = 7.3, p<0.05). Regarding T_4_ concentrations, no significant change was found in any group, (**Figure 4E**). In adult rats, HFD feeding led to an increase in T_3_ levels compared to controls, but only after 4 weeks of dietary exposure (**Figure 5D**). A two-way ANOVA showed a significant effect of time (F_(1,11)_ = 30.68, p<0.05) and diet (F_(1,11)_ = 4.8, p=0.05). T_4_ and TSH levels did not change in adults (**Figures 5C, E**).

### Corticosterone and leptin serum levels

We did not observe changes in serum CORT levels in any group of animals exposed to HFD (**Table 3**). In contrast, leptin levels in HFD-J12w increased by 79% relative to the C-J12w group (**Table 3**). Moreover, circulating leptin concentrations were 186% higher in HFD-J12w and 181% higher in HFD-A12w groups compared to their respective counterparts exposed to HFD for only 4 weeks (**Table 3**). Two-way ANOVA for juveniles showed significant effects of diet (F_(1,20)_ = 9.7, p<0.001) and time (F_(1,20)_ = 28.8, p<0.0001). For adults, a significant effect of time was observed (F_(1,18)_ = 8.3, p<0.01).

**Table 3:** Leptin and corticosterone serum levels.

Juveniles					Adults			
4w			12w		4w		12w	
Hormone	Control	HFD	Control	HFD	Control	HFD	Control	HFD
Corticosterone	156.8 ± 79	55.5 ± 17	144.4 ± 72	198.3 ± 67	131.4 ± 36	72.8 ± 17.4	269.2 ± 22.5	234.5 ± 15
Leptin	5375 ± 707	8722 ± 1556	1325 ± 2173	24944 ± 3278*^#^	7786 ± 2504	7431 ± 2205	11491 ± 2959	20876 ± 3751^#^

Leptin and corticosterone serum levels in control and high-fat diet (HFD) conditions. Data presented as the mean ± S.E.M and expressed in ng/mL of serum, (n=4-6/group). Two-way ANOVA statistical differences are denoted by *p < 0.05 vs. respective control and p< 0.05 vs. HFD-4w of the same age.

### IL-1β expression in the hypothalamic paraventricular nucleus

**Figure 6:**
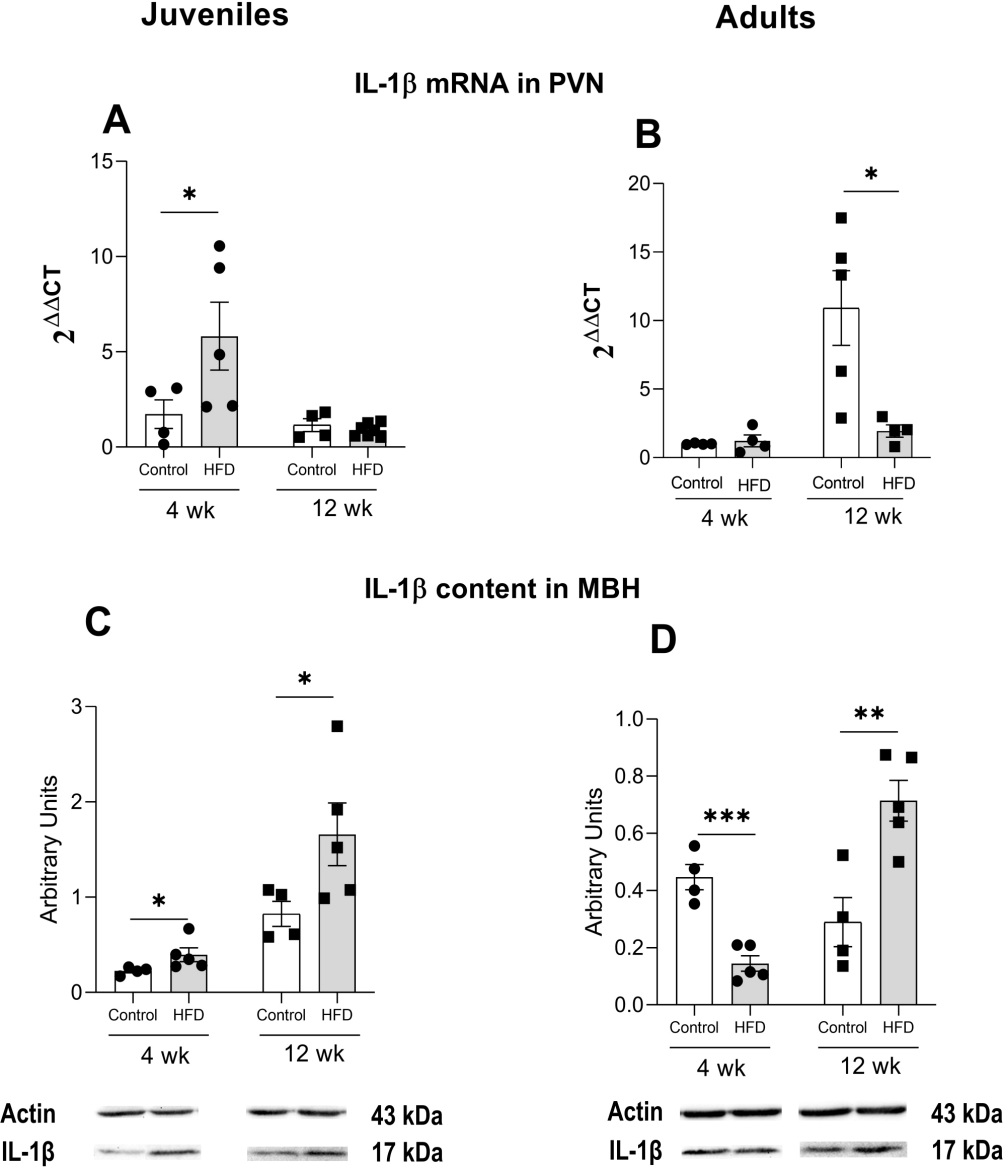
IL-1β mRNA and protein content. Gene expression was measured in the hypothalamic paraventricular nucleus (PVN) of juvenile **(A)** or adult rats **(B)** s. IL-1β protein content in the medial basal hypothalamus (MBH) of juvenile **(C)** and adults **(D)** rats. Animals were fed either a control diet or high fat diet (HFD) during 4 or 12 weeks. Results are presented in arbitrary units as the mean ± S.E.M., n=4–6 rats/group. *p<0.05, **p<0.01, ***p<0.001 between depicted groups.

To investigate possible mechanisms by which HFD intake influences the HPT axis and its relationship with neuroinflammation, we assessed the expression of IL-1β in the PVN and its content in the MBH. IL-1β is an important marker of pro-inflammatory processes (**Figure 6**).

PVN IL-1β mRNA expression increased more than 3-fold in juvenile rats after 4 weeks of HFD administration compared to controls. However, this effect was no longer observed after 12 weeks (**Figure 6A**). A two-way ANOVA showed a significant effect of duration (F_(1, 16)_ = 7.989, p<0.05) and a significant interaction between diet and duration (F_(1, 16)_ = 5.016, p<0.05). Similarly, in adult rats, HFD induced a marked increase, approximately 10-fold, in IL-1β expression after 4 weeks, but no significant change was detected after 12 weeks compared to controls (**Figure 6B**). In adults, the two-way ANOVA also showed significant effects on diet (F_(1, 13)_ = 10.13, p<0.01), duration (F_(1, 13)_ = 6.8, p<0.05) and interaction (F_(1,13)_ = 7.5, p<0.05).

IL-1β protein levels in the MBH were elevated in all HFD-fed groups, except for adults after 4 weeks of diet exposure (**Figures 6C, D**). Specifically, HFD-J4w rats showed a 74% increase (t(7) = 2, p<0.05), HFD-J12w rats exhibited a 2-fold increase (t(7) = 2.1, p<0.05), and HFD-A12w rats demonstrated a 2.5-fold increase vs. respective controls (t(7) = 3.8, p<0.01).

## Discussion

Our results showed that both age and duration of HFD exposure differentially affected b.w. gain, adiposity, HPT axis activity, and found that juveniles groups were the most affected. These results highlighted the complexity of neuroendocrine adaptations and the risk of being impaired when high-fat feeding is prolonged mainly in adolescence. Results gain relevance by the higher long-term consumption of HFD that in Western societies has been increased that has contributed to obesity development and its related disorders such as, metabolic syndrome, diabetes, and hypertension (26, 27).

We found that 4 weeks of HFD feeding was insufficient to alter HPT axis activity or to increase adiposity in either juvenile or adult rats. However, juvenile animals did show an increment in b.w. gain, which might be due to their high growth rate. Energy intake thus, could be redirected towards muscle protein synthesis rather than to lipid accumulation (28).

The observed HPT axis activation at both ages was consistent with diet-induced obesity models (7) and supported by their elevated PVN TRH expression along with absence in MBH peptide accumulation, implying TRH active release. This was confirmed by the high circulating T_3_ levels these groups showed. Such upregulation of the HPT axis is expected under conditions of positive energy balance, as the oxidation of dietary fats may be enhanced in HFD-4w juveniles and adults (29), potentially offsetting fat accumulation despite early signs of neuroinflammation.

Leptin, a positive regulator of TRH transcription, was unlikely the factor driving the PVN TRH upregulation, since its circulating levels did not increase in either 4-week HFD groups. Despite the observed elevated T_3_ serum concentration in 4-week-HFD groups, which normally suppress TRH, the PVN TRH mRNA did not decrease, which suggested that HFD-induced factors counteracted this negative feedback. For example, elevated free fatty acids (FFAs) from HFD intake may play a role by modulating hypothalamic signaling pathways. Mechanisms such as AMPK inhibition and endoplasmic reticulum stress may collectively promote TRH expression overriding classical feedback inhibition (30–33).

Notably, after only 4 weeks of HFD exposure, the enhanced hypothalamic IL-1β levels were insufficient to suppress PVN TRH mRNA synthesis (34). In this context, IL-1β–mediated suppression of TRH expression might require more prolonged or severe neuroinflammation to override these energy surplus–driven stimulatory inputs to TRH neurons.

When HFD intake was prolonged for 12 weeks, juvenile rats exhibited a deceleration of the HPT axis activity, suggesting that young animals are particularly vulnerable to the effects of long-term HFD exposure. Their HPT axis dysregulation might favor an impaired adaptation to a sustained positive energy balance, contributing to their increased b.w. gain and adiposity index.

As TRH release stimulates its own transcription in the PVN (35), the reduced TRH mRNA expression observed in HFD-J12w rats was accompanied by TRH accumulation in the MBH, while in adult animals, the HPT axis still appeared functional. Together, these data pointed to an age-dependent vulnerability of the HPT axis, with juveniles showing a limited adaptive capacity to prolong HFD intake.

HPT axis changes suggested that 12w-juveniles may have developed hypothalamic leptin resistance, consistent with their high blood levels, which has already been described after prolonged HFD exposure (7). Normally, elevated leptin levels enhance TRH expression in the PVN (36) however, despite high circulating leptin concentration, TRH mRNA levels declined. This indicates an impaired leptin-TRH signaling pathway, likely arising from long-term HFD intake and the increased adipose tissue. The resulting leptin resistance may have contributed to a blunted HPT axis response, to its inability to mount a compensatory response to the HFD-induced positive energy balance, facilitating lipid accumulation and exacerbating b.w. gain in juvenile animals.

Additionally, since serum corticosterone levels remained unchanged, it is unlikely that the observed downregulation of TRH synthesis was mediated by glucocorticoids, known inhibitors of TRH expression (37). This finding suggested that, in this context, HFD did not function as a physiological stressor.

Regarding neuroinflammation, this group (HFD-J12w) showed increased MBH IL-1β content, contributing to neuroinflammation onset. Although elevated IL-1β was already detectable at 4 weeks, its functional impact became evident only after prolonged HFD exposure, when both TRH transcription and release were downregulated. This suggested that HFD-induced IL-1β content might play a role in suppressing TRH expression and release, impairing HPT axis function (34). Supporting this notion, recent findings show that HFD-induced palmitoylation of PKC-δ in neuroendocrine microglia activates inflammatory pathways that disrupt TRH release (38).

It was striking that in juveniles, the long term HFD exposure was decisive to develop leptin resistance affecting the HPT axis activity. This could be explained by the combination of the sustained elevated leptin levels along with the IL-1β-mediated neuroinflammation that were only observed in 12w and not in 4w-HFD juvenile rats.

The high content of saturated fatty acids from HFD activates proinflammatory cytokines and toll-like receptor (TLR) when the intake is longer than 4 weeks. Consequently, the activity of the IKK β/NF-κB signaling pathway enhances in the hypothalamus (39), and in turn, upregulates the expression of the suppressor of cytokine signaling 3 (SOCS3), a well-known negative regulator of the leptin signaling cascade (40). The high levels of leptin in HFD juvenile rats along with the resulting inhibition of leptin signaling might contribute to the decreased TRH expression observed in 12 but not in 4w animals. Also, by acting synergistically with the enhanced IL-1β content, the hyperleptinemia blunted HPT axis responsiveness, promoting lipid accumulation and b.w. gain under chronic HFD exposure.

HFD-A12w rats exhibited increases in b.w. gain and adiposity similarly to those observed in juveniles. However, unlike HFD-J12w animals, the adult HPT axis remained active and appeared functionally adapted to the diet. TRH expression in the PVN was elevated, and instead of accumulated in the MBH, TRH content tended to be decreased. Moreover, TSH and T_3_ serum levels did not change, supporting the preservation of HPT axis output. Interestingly, despite elevated IL-1β levels in the MBH, PVN TRH mRNA expression was not downregulated, suggesting that in adulthood, the HPT axis is less vulnerable than in juveniles to the suppressive effects of hypothalamic IL-1β content.

Notably, the decrease in IL-1β MBH levels in HFD-4w adults contrasted with the increased content after 12 weeks of HFD exposure. This temporal pattern suggested that IL-1β-mediated neuroinflammation needs to be sustained to effectively disrupt TRH transcription, as observed in juveniles. Age-related differences in neuroendocrine responsiveness have been reported, with younger animals showing greater vulnerability to inflammatory stressors (19, 41). These findings highlighted that the adult hypothalamus is more resilient than that of younger animals to long-term HFD, likely due to maturational differences in immune-to-endocrine feedback sensitivity and central inflammatory tone.

Another difference between juveniles and adults after 12 weeks of HFD exposure was that BAT weight increased only in the younger animals, suggesting an age-dependent difference in thermogenic adaptation. In juveniles, BAT expansion likely represents a compensatory mechanism to manage excess energy intake through adaptive thermogenesis, thereby helping to limit b.w. gain. In contrast, HFD-A12w rats did not exhibit BAT hypertrophy, indicating a reduced capacity to activate thermogenic pathways despite maintaining an active HPT axis. This blunted response was reflected in their greater b.w. gain and higher adiposity index, emphasizing that endocrine activation alone may be insufficient to prevent lipid accumulation in the absence of peripheral thermogenic effectors such as BAT (42). Elevated TRH expression in the PVN can enhance sympathetic output to BAT, stimulating thermogenesis and fatty acid oxidation via β3-adrenergic receptor activation, which is also associated with high uncoupling protein 1 (UCP1) expression (43–45). In contrast, adults lacked BAT hypertrophy, reflecting a reduced capacity for thermogenic compensation and contributing to their higher adiposity despite preserved HPT axis activity.

The sample size of our study might limit the strength of our results since it is rather small for some determinations; however we significantly observed that prolonged HFD exposure, rather than short-term intake, drives lipid accumulation across age groups, even when b.w. gain increases are less pronounced. This reinforces the view that adiposity is a more sensitive and reliable indicator of diet-induced metabolic disruption than b.w. alone (46). Notably, after 12 weeks of HFD, both juvenile and adult rats exhibited increased b.w. gain and adiposity, despite their preserved or even elevated hypothalamic TRH synthesis, particularly in adults.

Our results suggested that HPT axis activation is not always sufficient to prevent fat accumulation during chronic high-fat feeding, highlighting a possible dissociation between neuroendocrine activation and effective metabolic compensation under sustained energy surplus. They also emphasized the importance of developmental stage in shaping neuroendocrine adaptations to HFD exposure. The long-term effects of HFD on the HPT axis, particularly when combined with neuroinflammation, may predispose individuals to impaired energy homeostasis and obesity. Future studies should explore the specific intracellular pathways and cell types involved in this regulation, including the role of cytokines beyond IL-1β, and the contribution of tanycytes, microglia, and other hypothalamic cell populations in sensing and integrating metabolic and inflammatory cues.

## Data Availability

The raw data supporting the conclusions of this article will be made available by the authors, without undue reservation.
